# PReS-FINAL-2239: Renal AA amyloidosis in a child with hyper-IgD syndrome and a novel MVK mutation

**DOI:** 10.1186/1546-0096-11-S2-P229

**Published:** 2013-12-05

**Authors:** S Fingerhutova, A Kolsky, D Rowczenio, H Lachmann, Z Vernerova, F Votava, P Dolezalova

**Affiliations:** 1 General University Hospital in Prague, Czech Republic; 2 University Hospital Kralovske Vinohrady, Prague, Czech Republic; 3 National Amyloidosis Centre, UCL Medical School in London, London, UK

## Introduction

AA amyloidosis may develop as a consequence of chronic inflammatory conditions including autoinflammatory diseases(AID). Mevalonate-kinase(MVK) deficiency(MKD) appears to be the least frequent underlying condition among monogenic periodic syndromes. Moreover, amyloidosis rarely manifests during childhood. We report a case of a small child in whom renal biopsy performed because of the cortico-resistant nephrotic syndrome revealed amyloid A.

## Objectives

To describe clinical manifestations, laboratory features and disease outcome in a patient referred for suspected periodic fever syndrome.

## Methods

Case report.

## Results

A 4-years old Caucasian girl with negative family history presented with features of nephrotic syndrome in 1/2011. Over the previous 2 years she has been suffering with recurrent episodes of unexplained fever with pharyngitis and lymphadenitis lasting 3 days in 2-4 weekly intervals and received a putative diagnosis of PFAPA (periodic fever, adenitis, pharyngitis, aphtae) syndrome. Despite the increasing frequency of febrile episodes over the last year investigations aimed at excluding monogenic fevers were not performed. In early 2011 her IgD was normal, IgA mildly elevated, serum amyoid A (SAA) fluctuated from normal to 200 mg/l. After the standard corticosteroid (CS) treatment of nephrotic syndrome had failed to induce remission after 6 wks, a renal biopsy was performed revealing amyloid A deposits in about 30% glomeruli. While genetic analysis was pending, Colchicine was added to the CS treatment followed by daily anakinra injections with good clinical response. After a laborious genetic screening to exclude mutations causing monogenic AID, following heterozygous variants in MVK gene were identified: Mutation V377I and a novel deletion in exon 5 C152WfsX6 (c.450_453delGGTG). The latter terminates the protein six amino acids after the deletion occurs, effectively making the protein shorter. Within 6 months of the treatment her proteinuria stabilised and there have been no signs of other organ involvement. Despite ongoing anakinra therapy she continues to have occasional febrile episodes with temporary increase of inflammatory parameters including SAA.

## Conclusion

MKD/HyperIgD-syndrome has been so far reported in only a few cases of AA-amyloidosis. Our patient has been the youngest one to develop this severe complication. The only other child reported so far was also a compound heterozygote carrying the genotype G326R/V377I. The long-term follow-up with careful SAA serial measurements will tell us more about the prognostic significance of the newly described MVK gene deletion. This case report also underlines the importance of careful differential diagnostic re-evaluation of children presenting with PFAPA phenotype in whom febrile episodes do not show a typical prolongation of afebrile intervals over the time.

## Disclosure of interest

None declared.

